# Portrayal of gender roles in Emirati television dramas: a content analysis

**DOI:** 10.3389/fsoc.2025.1506875

**Published:** 2025-02-05

**Authors:** Viola Gjylbegaj, Ahmed Farouk Radwan

**Affiliations:** ^1^Department of Mass Communication, Abu Dhabi University, Abu Dhabi, United Arab Emirates; ^2^College of Communication, University of Sharjah, Sharjah, United Arab Emirates

**Keywords:** gender roles, Emirati media, socio-cultural evolution, drama, cinema

## Abstract

This research investigates the portrayal of gender roles in Emirati television dramas and movies, contextualizing them within the evolving socio-cultural landscape of the United Arab Emirates (UAE). The study conducts a comprehensive analysis of Emirati dramas and movies, categorizing prevalent gender role trends and their alignment with Emirati culture. Through content analysis, the research identifies four primary patterns: the ratio of male to female characters, gender role diversity and plot-power, themes relating to culture and religion, and the intensity of gender role manifestation with external factors. Findings reveal a predominant focus on male characters in Emirati media, reflecting traditional values where men are depicted as providers and leaders, while women are often relegated to caretaking roles. Implications of the research highlight a gradual shift toward gender equality in the UAE, evidenced by the increasing participation of women in the workforce. However, entrenched cultural values continue to influence societal expectations, perpetuating certain gender stereotypes. The study underscores the importance of media literacy in fostering critical analysis and demands for more nuanced female representation in Emirati media. Recommendations are proposed for media producers and regulators, to collaborate in promoting gender-sensitive content and fostering social change. By embracing evolving gender dynamics while preserving cultural heritage, the UAE has the potential to emerge as a global exemplar of contemporary society, championing diversity and inclusivity on a regional and international scale.

## 1 Introduction

Gender roles, defined as “the social and cultural expectations for how people should behave according to their assigned gender” (United Way NCA, 2023), have been integral to societal structures since the dawn of civilization. These roles are deeply embedded in cultures worldwide, yet their manifestations vary significantly. In the context of Emirati society, understanding the impact of gender roles and their representation in media is essential for grasping broader social dynamics. Middle Eastern films often portray negative stereotypes by focusing on themes of women’s oppression. However, these depictions are typically intended as critiques of the cultural and religious conditions that shape these societal dynamics, particularly in their treatment of women (Atakav, 2016). Women are often depicted through the lens of deep-rooted stereotypes and patriarchal values, confined to roles that uphold traditional gender norms. They are commonly portrayed as passive figures, either embodying male fantasies or representing cultural ideals of virtue. Such simplistic and reductive portrayals fail to capture the complexity of women’s lives, perpetuating gender inequalities and hindering progress toward meaningful social transformation (El Fellak and Ennam, 2024).

This research focuses on analyzing gender roles in Emirati media, with a particular emphasis on television dramas and movies. By conducting a content analysis, the study investigates how gender roles are depicted and developed within the context of Emirati media. The analysis aims to identify prevailing trends, patterns, and discrepancies in gender portrayal, highlighting the representation of both men and women in various roles, relationships, and professions.

While the research primarily focuses on the content analysis of Emirati media, the broader context of gender roles in the UAE society is also considered, as it provides the cultural foundation that shapes these media representations. The study examines how traditional and cultural factors influence gender expectations and norms within Emirati society, contributing to a deeper understanding of how gender dynamics are depicted in local media. The study aims at evaluating the complexities of gender roles in Emirati media and identify areas for improvement in promoting gender diversity while respecting the UAE’s traditional values. By providing a comprehensive analysis of gender dynamics in Emirati media, this research contributes to the ongoing conversation about gender equality and representation in the region. It offers actionable recommendations for media producers, regulators, and activists to collaboratively foster gender-sensitive content and support social change aligned with the UAE’s evolving societal norms.

## 2 Literature review

The portrayal of gender in Arab drama is a multifaceted issue, reflecting broader societal norms and cultural contexts. This summary explores the representation of gender roles in Arab drama, highlighting key findings from various studies (Kharroub and Weaver, 2014). Women are significantly underrepresented in Arab drama. They are less likely to have recognizable jobs and are often depicted in sex-typed occupations and settings. Female characters are frequently confined to traditional roles, such as caretakers or those associated with family disputes, reinforcing gender stereotypes (Edam et al., 2024). This is particularly evident in the portrayal of female lawyers in Lebanese and Egyptian cinema, where they are often depicted in caretaker roles and defined by their relationships with male protagonists (Möller, 2023).

### 2.1 Gender representation in media

The representation of gender in media is a powerful mechanism through which societal norms and expectations are reinforced. In this general narrative literature review, studies on gender stereotypes in the Middle East, such as those by Khalil and Dhanesh (2020), reveal that although there is increasing awareness about gender equality, television advertising still tends to reflect traditional gender roles, positioning women predominantly in domestic and passive roles. El-Sheikh (2023) highlights how Arab TV commercials are increasingly portraying empowered female figures, though traditional gender expectations remain embedded in many advertisements. In contrast, global advertising trends like femvertising challenge these stereotypes by promoting empowering portrayals of women (Åkestam et al., 2017). However, the impact of these advertising strategies remains mixed, and in many contexts, including the UAE, they are still a work in progress.

The portrayal of women in Gulf dramas, as discussed by AlAli (2018), is also evolving, though gendered expectations still prevail. These portrayals often reflect a tension between traditional values and modern aspirations, showing women in a variety of roles, from housewives to career women. Allagui and Al-Najjar (2018) explore the link between women empowerment and nation branding in the UAE, emphasizing how media strategies are intertwined with national development goals and how the portrayal of women is used to project a modern and progressive image globally.

In comparison, Egyptian drama has long focused on women’s issues, but the treatment of these issues can often be simplistic or stereotypical. Alsayad (2023) and Alwazan (2020) analyze how Egyptian television portrays women, arguing that although women’s social roles are central to the narratives, they are often framed within narrow expectations. This focus on women’s issues in Egyptian media has parallels in Emirati media, where women are increasingly shown as central figures in their own right, yet still, their roles remain closely tied to family and domesticity.

Meanwhile, international research further highlights the cultural and psychological implications of media portrayals. Studies by Brinkman et al. (2015) show how college women reflect on media representations of empowerment, noting that these portrayals can both inspire and limit women’s self-image. In a similar vein, Brún et al. (2013) explore how the Irish media portrays gender and obesity, revealing how stereotypes of responsibility and health are gendered, which can be compared with the way media in the UAE often places disproportionate emphasis on women’s physical appearance.

Globally, media content continues to evolve, reflecting changing attitudes toward gender and social roles. In the UAE, television shows and films are increasingly featuring empowered women, although often within a framework that blends traditional values with modern portrayals. Chen (2023) discusses how female body image is portrayed in social media, and the shift toward more authentic and inclusive representations of women in media is starting to be reflected in UAE-based media as well.

### 2.2 Cultural and workplace dynamics

Gender representation in the workplace, particularly within professional sectors, has a direct influence on how men and women are portrayed in media. Studies like those by Patterson et al. (2020) illustrate how gender gaps in fields like STEM persist in the UAE, and these gaps are reflected in the media, where women are often underrepresented in professional roles. In parallel, Lee et al. (2023) discuss how gender biases influence the perception of UAE Public Relations professionals, with women often facing additional challenges due to cultural expectations.

The broader impact of gender roles in the workplace is explored by Abaker et al. (2022), who discuss the barriers that women face in private organizations in the UAE. These obstacles in professional settings are mirrored in media portrayals, where men tend to hold leadership roles, while women are depicted in supporting positions. While Emirati media continues to depict men in dominant leadership roles, government policies increasingly emphasize gender equality (PCHR, 2019). Efforts such as equal pay legislation and national strategies for women’s empowerment are gradually influencing workplace dynamics, a shift that is yet to be fully reflected in media portrayals.

### 2.3 Social media and gender

Social media platforms serve as a double-edged sword for gender representation. While they offer opportunities for women to voice their experiences and challenge traditional gender norms, they also perpetuate stereotypes. Snoussi (2024) discusses the complex role of social media in the MENA region, noting that while it can be empowering, it also reinforces certain gendered expectations, particularly around appearance and behavior. This dynamic is especially evident in the UAE, where social media has both enabled young women to express their agency and simultaneously subjected them to scrutiny based on traditional gender norms.

Storie and Marschlich (2022) explore how young Emirati women engage with the representation of female politicians on social media, highlighting the tension between admiration for political figures and criticism of their curated public personas. This study underscores the importance of authentic representation, and the challenges women face when navigating traditional and modern societal expectations.

### 2.4 Historical and global perspectives

The historical context of gender roles provides important insights into contemporary media portrayals. Koch and Kirleis (2019) discuss how gender transformations in prehistoric and archaic societies inform current gender dynamics, suggesting that the portrayal of gender in media cannot be understood without acknowledging the deep cultural roots of these roles. This historical lens helps frame current discussions on gender representation in the UAE and other parts of the Middle East, where media is caught between tradition and modernity.

In contrast, international perspectives, such as those by Hudson (2020a), Hudson (2020b) and Kozma (2016), suggest that as globalized media interacts with local cultures, there is a dynamic exchange that influences gender portrayals. Hudson (2020a) and Hudson (2020b) specifically discusses how Dubai’s cinematic landscape reflects this tension between cosmopolitanism and traditional Arab values, offering a unique insight into the complexities of gender representation in the UAE. Similarly, the study by Park (2012) on mediated intergroup contact suggests that media portrayals play a key role in shaping societal perceptions of gender and can either reinforce or challenge existing stereotypes.

### 2.5 Gender and the future of media

The future of gender representation in media looks to be increasingly shaped by both technological advancements and shifting societal attitudes. As discussed by Radcliffe and Lam (2018), social media has played an important role in reshaping the gender landscape in the Middle East, offering opportunities for more progressive gender portrayals while simultaneously creating new challenges. This tension is mirrored in the portrayal of gender roles in traditional and digital media formats, highlighting the need for greater diversity in how gender is represented.

The increasing representation of women in leadership roles in both the media and society offers a more nuanced view of gender roles. Media must evolve alongside these shifts to reflect the complexity and diversity of gender identity, challenging traditional norms and contributing to greater societal change.

## 3 Methodology

This study uses the qualitative approach by employing content analysis to examine gender role portrayals in Emirati media, specifically focusing on television dramas and films produced in the UAE over the past decade. The selection of media for analysis included popular television series, films, and online content, chosen based on factors such as cultural relevance, viewer ratings, and their representation of gender roles. Each piece was carefully selected to ensure a broad and representative sample of Emirati media that reflects the cultural and societal dynamics of the country, offering insights into the evolving portrayal of gender within the context of local norms and global influences.

A detailed content analysis was conducted to identify recurring themes and patterns in the depiction of gender roles. This involved coding scenes and character interactions to assess the frequency and context of both traditional and progressive gender representations. Specific criteria were developed to evaluate various aspects such as character profession, family roles, behaviors, and dialogues. The analysis focused on identifying the representation of male and female characters, their roles within the narrative, and the alignment of these roles with societal norms and expectations. The authors defined 9 variables to analysis including Predominantly Shown Gender(s), Character Traits, Occupations, Relationships, Decision-making Power, Religious Values vs. Cultural Values, Frequency of Traditional vs. Non-Traditional Gender Roles, and Intersectionality of Gender Roles with Other Factors (socio-economic status, time period, etc).The findings from the content analysis were interpreted within the context of Emirati culture, considering cultural norms, values, and societal expectations in the UAE. This interpretation also considered the influence of historical, social, and economic factors on gender roles and their representation in the media. The study also proposes areas for future investigation to further understand gender dynamics in the UAE and similar cultural settings.

### 3.1 Selection of television dramas and films

In recent years, dramatic and cinematic production in the UAE has surged, with increased diversity and improved quality in Emirati productions. Government support and growing cultural interest drive this growth across film and television. Television drama flourishes with varied series, enriching viewer options and involving local talent (Hudson 2020a; Hudson 2020b). The film industry sees a rise in domestic movies and attracts international productions, benefiting from the UAE’s infrastructure and diverse scenery. Emirati works cover heritage, societal issues, and human stories, while international collaboration enhances the industry’s global presence. This dynamic growth reflects the UAE’s commitment to a vibrant media landscape aligned with its identity and values (Hudson 2020a; Hudson 2020b).

For this study, we focused exclusively on Emirati dramas to ensure an accurate depiction of local culture. To identify current and influential movies and TV series, we consulted with Emirati individuals and experts. Based on their recommendations, we selected one movie and four popular TV series for an in-depth analysis as they discussed social issues. These selections encompass a variety of genres and target diverse audiences, from children to adults, and have been widely accessible across multiple platforms. The authors analyzed the drama from https://adtv.ae/ar.

### 3.2 Coding scheme table

**Table 1:** 

	Categories								
Television shows/movies	Genres	Predominantly shown gender(s)	Character Traits	Occupations	Relationships	Decision-making power	Religious values vs. cultural values	Frequency of traditional vs. non-traditional gender roles	Intersectionality of gender roles with other factors (socio-economic status, time period, etc)
Al Kameen (movie)	Drama, War, Action	Men	Brave, Respectable, Emotional	Only male characters: Soldiers, Commanders, and Military Personnel	Comrades and friends in War (Main Characters); Interaction with enemies in war; Families at home (Wife and Children)	Men	Islamic Values and Cultural Values: All characters are heavily implied to be Muslim: fighting in the name of Allah SWT and for their country.	Mainly Traditional Gender Roles: features men fighting on the battlefield (adheres to men’s gender stereotypes of fighting for their country)	Male Gender Roles Intersect with War; time period in the movie (2018) showcases tension with the enemy with strengthens the traditional Emirati gender roles of men
Khash’ie Nash’ie (television show)	Comedy	Men with some female characters	Dramatic, Funny, witty	Only male characters: changing occupations depending on the plot (Store owner, Gas Station, Driver)	Friends (Main Characters); Interactions with side characters (Managers, Principal, Doctors)	Men	Cultural Values: The two main characters are implied to be Muslim; however, the story is more based on cultural change	Traditional Gender Roles: story is mainly about the antics of two men who try to get a long-term job (adheres to men’s gender stereotypes of needing a job)	Male Gender Roles Intersect with Modernization: the men residing in 1960s to 1970s struggle to integrate modern technology into their village life, while trying to find success in their occupations
Simple Life (television show)	Comedy	Women with some male characters	Dramatic, greedy, competitive	Male and female characters: changing occupations depending on the plot (Female: incense seller, medicine field, restaurant owner) (Male: Unspecified work, unemployed)	Family (Mother, Father, Son, Daughter); Extended family (Grandfather); Interactions with side characters (Rivals, Suitors, Daughter’s Ex-Husband, Son-in-Law)	Women	Cultural Values: characters are implied to be Muslim, but there is not direct effect of religious values as much as there are cultural affects	Mainly non-traditional gender roles: features a main female character who is working to provide for her family, but marriage and children are commonplace for the women (deviates from women’s and men’s stereotypes, but still portrays tradition)	Female gender roles intersect with socio-economic status: the main female character has to provide for her family and make money, while also caring for traditional aspects such as her daughter’s marital issues.
Shabiyat Alcarton (television show)	Animation, comedy, satire, sitcom	Men and women	Funny, clever, witty	No specified occupations	Friends (main characters)	Men	Islamic values and cultural values: present in ways of dressing, but neither are heavily mentioned	Non-traditional gender roles: does not differentiate between male and female characters; shows them as equals in conversation	Male and female gender roles intersect with no specific factor: the show is episodic and does not heavily focus on gender dynamics.
Khosa Bousa (television show)	Animation, comedy	Women with some male characters	Intelligent, Interesting, Funny	No Specified Occupations	Family (main characters: grandparents, parents, children)	Women	Islamic values and cultural values: expressed through clothing and tradition.	Mixture of both: shows that men must have jobs and women must have household responsibilities but shows female characters going on adventures and learning.	female gender roles intersect with no specific factor: the female characters go on episodic adventures where they get in trouble or learn something new, but gender role intersection is not prominently noticeable.
Al-Boum (television show)	Drama and Action	Men with some female characters	Dramatic	Sailor	Family and friends	Men	Cultural values: courage, danger facing, and challenge.	The traditional role of the man in the image of the victorious hero facing dangers and difficulties	It focuses on the leading role of men in society and being the seeker of work and livelihood
Khattaf (television show)	Sports, Action, Drama	Men with some female characters	Dramatic	Athlete	Friends	Men	Cultural values: challenge, and determination	The traditional role of the man in the image of the victorious hero facing dangers and difficulties	It focuses on a man’s ability to achieve success, face challenges, and reach the international level
Wdima & Halima 3 (television show)	Comedy	Women with some male characters	Comedy	Housewife	Family and friends	Women	Cultural values: conflict ‑‑‑ neighborhood	The conflict between neighboring women is a traditional image of women that assumes that they are in a state of jealousy and envy toward their neighbors. The series deals with many social issues in a comedic framework	It works to show required social values in a comedic form through the conflict between two neighbors
Algozoh (television show)	Thriller	A mixture of men and women	Villagers		Family and friends	Men and women	Cultural values: confronting conspiracies	It is based on folk tales and legends that show The struggle with the injustice and corruption that spreads in the village	It focuses on the role of women in the success of their family and supporting their husbands. Although it was based on a myth, it showed women in the role of perseverance.
Beat Apona (television show)	Social	Equal roles	Social	Family	Family	Men and women	Cultural values: brotherhood values	It focuses on the social aspect and the importance of agreement between siblings, even if differences arise between them due to inheritance	In a comedic setting, three separated siblings are forced to live together in one house that they inherited from their father after he recommended not selling it. Through their life together (two boys and a girl), they discover the importance of the family and the values of brothers.

## 4 Data collection

### 4.1 The series “Al-Boum” (2024)

The series “Al-Boum” produced in 2024 tells a heroic story represented by an ambitious and stubborn young man who challenges the sea and its might to achieve his dream. He navigates the sea with his wooden boat under challenging conditions, in search of pearls, fish, trade, wealth, and glory. The series is set during World War II, between the years 1939 and 1945, where the young sailor leads a team of fishermen and sailors, venturing into the Indian Ocean. They face towering waves, strong winds, the perils of war, internal crew conflicts, and the schemes of competitors.

### 4.2 The series “Khattaf”(2024)

The series “Khattaf” produced in 2024 revolves around a dramatic and suspenseful framework, shining a light on the story of a young Emirati man aspiring to become a hero in combat sports. The events unfold within a dramatic context, as the protagonist strives to become a champion in combat sports, but he faces challenging and risky obstacles along his athletic journey.

### 4.3 The series “Wdima & Halima 3” (2024)

The events of the comedy series take place in the 1980s in one of the popular neighborhoods, between the house of “Halima” and the house of “Wadima,” revolving around these two characters and the existing animosity between them, resulting in disputes over trivial reasons, in addition to their attempts to plan pranks to retaliate against each other. The difference in temperament and personality of each of them affects the type of pranks and their reactions. Halima is a spinster, illiterate, who loves raising livestock, while Wadima, on the other hand, is a married woman with multiple marriages, literate, who knows reading, writing, and arithmetic, and has her own trade. Amidst this war between them, an innocent and undisclosed love relationship arises between “Salem,” the son of “Faraj,” and “Asma,” the daughter of “Wadima,” which develops but clashes with the enmity between the two neighbors.

### 4.4 The series “Algozoh” (2024)

The series delves into a traditional setting, narrating the story of a popular woman who tirelessly attempts to cure the villagers through traditional folk remedies in the old-fashioned way. She endeavors to assist them in various ways amidst the difficult circumstances they live in, while also dealing with the problems and conflicts that arise between her husband and his archenemy. The series addresses social issues and struggles concerning the conflict over money for survival and employment.

### 4.5 The series “Beat Apona” (2024)

The series tells the story of three siblings who inherit a luxurious villa from their father in the heart of Jumeirah city. They decide to sell it to settle their debts and improve their lives, but they encounter a surprise when they discover their father’s will, which prohibits the sale of the villa entirely. The siblings are forced to live together in the villa, despite their personal differences and lifestyles. A journey filled with comedic and dramatic situations begins as they learn to adapt to each other and manage the affairs of the villa.

### 4.6 Al Kameen (2021)

Released on November 25, 2021, Al Kameen (The Ambush) is a drama, war, and action movie produced by AGC Studios and Image Nation Abu Dhabi. Set during the Yemen war in 2018, it narrates the story of three Emirati soldiers ambushed and trapped in enemy territory, while their comrades devise a rescue plan. The film predominantly showcases male experiences during the war, focusing on their daily activities in the Mocha Base camp, such as morning exercises and watching football. The soldiers are also depicted dealing with the harsh realities of war, including tension-filled arguments and reminiscing about their families. On the battlefield, the men face danger, victory, and death, with most characters being soldiers, commanders, or military medical personnel. The film emphasizes the camaraderie among the soldiers and their hierarchical relationships, with decision-making power primarily in the hands of higher-ranking male officers.

### 4.7 The series “Khash’ie Nash’ie” (2020)

Khash’ie Nash’ie, a comedy series released on April 24, 2020, by Abu Dhabi Television, is set in the 1960s and explores the daily lives and challenges of characters in modern society while upholding traditions and cultural norms. The series portrays various aspects of Emirati society, including cultural traditions, family, and social norms, alongside developments in education, health, and infrastructure. The two main male characters deal with their issues in a dramatic and comedic manner, often changing occupations depending on the episode’s plot. Relationships in the series include friendships and interactions with side characters, with decision-making predominantly in the hands of the male characters, leading to conflicts that drive the plot.

### 4.8 The series “simple life” (2019)

Simple Life, a comedic TV drama series made in 2017, depicts the everyday life of an Emirati family, addressing themes of family duty, community, marriage, temptations, and conflicts. The characters exhibit dramatic mannerisms and are portrayed as greedy and competitive. The series highlights the evolving gender dynamics in Emirati society, with female characters like Ruqia taking on various jobs to support their families. The drama, primarily from a woman’s perspective, shows women making significant decisions for their families and themselves.

### 4.9 The series “Shabiyat Alcarton” (2006)

Shabiyat Alcarton, an Emirati animated sitcom first broadcast in 2006 during Ramadan on Sama Dubai, has over 18 seasons and continues to air. The series, featuring comedy and satire, revolves around the lives and traditions of Middle Eastern families and individuals living in Dubai. The main character, Shambeeh, and his friends encounter various comedic situations. The show treats its male and female characters equally, with both genders playing significant roles in the plot. However, decision-making power often lies with the main character, Shambeeh.

### 4.10 The series “Khosa Bousa” (2009)

Released in 2009, Khosa Bousa is a 3D animated comedy series that humorously portrays the daily lives of an Emirati family. The show challenges traditional gender roles by depicting women like Amina Namki as knowledgeable and capable, countering stereotypes of female intelligence. Female characters such as Hamama, Nafeesa, and Sheikha navigate familial dynamics and societal expectations, while male characters are shown engaging in work, socializing, and traditional events. Female characters are central to the show’s plot, making critical decisions that drive the storyline.

In our content analysis, four main patterns emerged across the selected media: the ratio of male to female characters, gender role diversity and plot power, themes relating to culture and religion, and the influence of external factors on gender role manifestation.

#### 4.10.1 Ratio of male to female characters

Male characters appear more frequently than female characters and often hold significant roles. In Simple Life and Khosa Bousa, despite focusing on women’s perspectives, men still play notable roles. Al Kameen and Khash’ie Nash’ie primarily focus on male characters, with female characters having minimal relevance. Conversely, Shabiyat Alcarton features a balanced representation of both genders, with character importance varying by episode.

In 2024, several Emirati series were produced featuring both men and women in leading roles. Although the focus on the successful protagonist was often attributed to men, as evident in the series “Al-Boum” and “Al-Khattaf,” where the protagonist in the former was a successful sailor who overcame challenges, and in the latter, a sports hero, with women typically playing the roles of lovers or wives. However, the series “Beit Abuna” addressed equal relationships between two brothers and their sister in social events revolving around inheritance. Similarly, in the fantasy series “Al-Jadu’ Al-Khayali,” the wife played a fundamental role in supporting her husband. On the other hand, the comedy series “Halima and Wadima” portrayed a comedic conflict between two neighbors. Hence, the successful protagonist in his work remains the one with a strong and persevering personality, who challenges the odds, typically revolving around men. Meanwhile, women are often depicted as supportive wives, sisters, or comedic characters involved in conflicts with their neighbors. This portrayal may no longer reflect the reality of the UAE, where women have proven their strength and affirmed their success.

#### 4.10.2 Gender role diversity and plot power

Shows like Simple Life, Khosa Bousa, and Shabiyat Alcarton display more diverse gender roles. Female characters in these shows are complex, balancing cultural traditions with modern responsibilities. In contrast, Al Kameen and Khash’ie Nash’ie focus predominantly on male experiences and decisions, with plot power centered around male characters. AlAli (2018) concluded that Emirati women perceive their portrayal in Gulf dramas as far from reality, depicting them as controlled by men and passive. In contrast, drama offers a more positive image than what is presented in dramas.

#### 4.10.3 Cultural and religious themes

Traditional values and cultural aspects, such as clothing and social norms, are prevalent in all analyzed media. Characters often embody Emirati cultural and Islamic values, balancing modernization with tradition. This is evident in Al Kameen’s portrayal of honorable soldiers and the cultural focus in Khash’ie Nash’ie, Simple Life, Khosa Bousa, and Shabiyat Alcarton.

#### 4.10.4 External factors influencing gender roles

External factors amplify traditional gender roles in some media. In Al Kameen, the battlefield context intensifies male leadership and bravery. In Khash’ie Nash’ie, modernization challenges traditional male roles. Simple Life juxtaposes traditional female roles with economic responsibilities. Shabiyat Alcarton and Khosa Bousa, being more character-driven and episodic, show less prominent engagement with external factors impacting gender roles.

## 5 Discussion

Our content analysis reveals a gradual but slow change in gender dynamics within UAE media, mirroring the broader societal and cultural shifts in the country. Shows like Al Kameen and Khash’ie Nash’ie predominantly focus on men’s experiences, whether in serious or comedic contexts, illustrating male-dominated spaces. In contrast, Simple Life and Khousa Bousa, which address women’s issues, do not enjoy the same level of focus on female-exclusive spaces. Men’s issues remain prevalent in these shows, reflecting the existing cultural norms in modern Emirati society.

In the UAE workplace, there is a “positive trend” toward “gender equality” (Abaker et al., 2022), with more women entering the workforce to establish themselves as career professionals. However, significant gaps persist. Our secondary research indicates that while men and women in PR are generally treated equally, a survey revealed that “female respondents expressed that they faced bias more than male participants (Lee et al., 2023). This bias often pertains to opportunities at work and is rooted in traditional values that expect women to uphold certain cultural norms. As women integrate into the workforce, these biases highlight the existing male-dominated nature of professional environments, a reality that is reflected in Emirati media.

The portrayal of gender roles in the analyzed dramas and movies influences current perceptions of gender dynamics. By featuring more women on screen and providing them with well-rounded characterizations—such as being funny in Simple Life, interesting in Khousa Bousa, or active participants in Shabiyat Alcarton—these shows help normalize the presence and importance of Emirati women in media. These female characters, who embrace and are proud of their traditional and cultural values while also being portrayed as individuals with distinct personalities, offer a more balanced and realistic representation of women.

As Emirati society continues to evolve, incorporating women more fully into the workforce, educational institutions, and other sectors, media representations will likely follow suit. This reciprocal relationship between media and societal norms suggests that as media becomes more inclusive and reflective of gender equality, it will continue to influence and shape cultural perceptions and practices in the UAE.

To conclude: in Emirati television dramas, gender roles often adhere to traditional values, portraying men as primary figures engaged in work, provision, and community leadership, while women are predominantly depicted as caretakers and homemakers. Conversely, international television dramas present a more diverse spectrum of gender roles and dynamics, challenging traditional norms. Notably, women in international dramas frequently occupy central roles as heroines or villains. For instance, The Crown provides a compelling portrayal of Queen Elizabeth II’s ascent to the throne of the United Kingdom. The series delves into the complexities of gender roles, showcasing the Queen’s journey as a woman assuming the highest position of authority in the Commonwealth. Throughout the narrative, the series underscores the challenges faced by her husband, Prince Philip, as he grapples with living in her shadow. Moreover, subsequent seasons depict how other characters, including her children, navigate similar struggles. This nuanced portrayal effectively highlights the inequality and hurdles encountered by women in positions of authority, emphasizing the significance of empowerment.

Despite significant advancements in gender roles in both the UAE and Western countries, Emirati media has yet to fully embrace the intricacies of female representation. By incorporating women’s perspectives more prominently in their narratives, Emirati media has the potential to catalyze profound changes in societal gender norms.

## 6 Implications and recommendations

The findings of our research suggest forthcoming shifts in gender equality, media literacy, and societal dynamics. Regarding gender roles, our investigation highlights a growing challenge to traditional norms, with increasing support for women’s empowerment in the UAE. As more women enter various sectors such as public relations, politics, and the military, there is a gradual representation of their presence in media portrayals, as evidenced by the dramas and movies analyzed. However, despite this finding toward diversified gender roles, societal emphasis on preserving cultural and Islamic values, such as modesty and traditional gender responsibilities, persists. For instance, while women are encouraged to work, they are still expected to fulfill household duties and child-rearing responsibilities, while men are tasked with providing for the family. Moreover, adherence to cultural attire remains an important aspect for both genders. Thus, while gender equality is increasingly favored over traditional roles, certain cultural facets will continue to endure due to Islamic and nationalistic convictions.

In terms of media literacy, there is a growing intention among Emiratis and UAE residents to critically evaluate local media against international standards. This finding signifies emerging discussions on gender diversity, particularly in terms of female representation. There is a notable demand for more nuanced and well-developed female characters, as exemplified by shows like Simple Life and Khousa Bousa, albeit with room for improvement in their character arcs. These shows serve as steppingstones toward portraying more complex female characters akin to the depth exhibited by male characters in productions like Al Kameen. Furthermore, with the UAE’s strategic utilization of female empowerment for “nation branding,” it is foreseeable that more individualistic female characters will be spotlighted in media narratives.

Additionally, our research suggests the potential for tangible social change, as shifts in the media landscape reflect evolving societal norms. While legislation promoting gender equality, such as equal pay in the public sector, has been enacted, there remains a need for concerted efforts to ensure more diverse and gender-sensitive content across media platforms. Collaboration between activists, lawmakers, and media producers is imperative to promote better gender representation while safeguarding the rights of all involved in production.

Our recommendations extend to media producers, regulators, and activists in the UAE. Media producers should establish guidelines that prioritize gender balance in content creation, fostering inclusivity across all aspects of production. Regulators should not only enforce existing policies but also introduce guidelines specifically addressing gender diversity in media. Inclusive projects should be prioritized, with regulators taking proactive measures to educate audiences on modern portrayals of gender roles. Activists play a crucial role in raising awareness and advocating for gender role changes through public campaigns and events, thereby influencing public opinion and fostering a conducive environment for addressing gender stereotypes.

The findings raise questions about the influence of the market, the desire for distribution, and the increase in viewership ratings, as it appears that the audience still seeks the brave male hero who triumphs over enemies and possesses a strong physique. The results also raise questions about the role of women in the drama industry and their active presence in the profession as authors, directors, or screenwriters, which could lead to a greater focus on women’s issues.

In comparison to Egyptian drama, which boasts the largest Arab production compared to other Arab countries, we find that distribution standards, the fame of artists, and the presence of women in the drama industry have led to the creation of numerous works that focus on women’s issues, with women often being the central focus of the work (Alsayad, 2023; Shemes, 2023) This has not prevented the continued existence of a type of bias in favor of men (Alwazan, 2020).

In conclusion, our research underscores the evolving landscape of gender roles in Emirati media and the potential for transformative change. By addressing gender stereotypes and promoting inclusive narratives, the UAE can pave the way for a more diverse and equitable society, aligning with its aspirations for progressive nation-building.

## 7 Conclusion

In conclusion, our research reveals a dynamic shift in gender roles within Emirati media as the UAE continues its trajectory toward modernization. As the country evolves, so too do its traditions and cultural values, reflecting a progressive stance toward gender equality. Our primary and secondary research indicates a clear vision toward the empowerment of Emirati women, driven by governmental initiatives to ensure their equal representation alongside men in various spheres of society. However, despite this progress, our comprehensive analysis and international comparisons underscore the lingering pace of change. Female characters in Emirati shows and movies often receive unequal treatment compared to their male counterparts, signaling a need for further evolution in media content to align more closely with the current societal norms. This highlights the imperative for collaboration among showrunners, producers, and policymakers to ensure gender-sensitive representation across all media platforms, including television, social media, and other outlets. By grappling with these gender dynamics, the UAE is poised to emerge as a trailblazer in fostering diversity within the MENA region. By embracing evolving social roles for both men and women while preserving its rich traditions and culture, the UAE stands as a potential beacon of contemporary society done right, setting a precedent for inclusive and equitable representation on a global scale. The limitations of this study were defined by analyzing a number of Emirati drama. Further research on this topic is required to explore additional variables, such as the nature of production, different social roles, and the impact of competition and marketing factors in the local and Gulf markets. In addition, the audience’s perspective and his evaluation about what Emirati drama presents can be studied, examining whether it reflects genuine social roles or portrays unrealistic models. The issues, themes, and characters addressed in drama are not solely tied to the culture or traditions of society but are also influenced by production factors, market demands, and the priorities of companies and media outlets involved in the entertainment industry and its distribution. Therefore, the focus on certain social roles or the portrayal of specific societal groups in drama is not merely a reflection of the writer’s perspective or cultural and societal influences but is also shaped by various economic and production-related factors.

## 8 Limitations and further directions

The scope of the study is limited to analyzing a selection of television series and a single film, based on the exploratory research conducted by the researchers. This paves the way for future studies to examine a broader range of productions, including radio and television dramas as well as cinema movies, across the UAE and the Gulf Cooperation Council (GCC) countries, many of which are witnessing significant social and cultural development particularly in Saudi Arabia.

## Data Availability

The raw data supporting the conclusions of this article will be made available by the authors, without undue reservation.
